# Quercetin Protects Human Thyroid Cells against Cadmium Toxicity

**DOI:** 10.3390/ijms22136849

**Published:** 2021-06-25

**Authors:** Francesca Capriglione, Jessica Maiuolo, Marilena Celano, Giuseppe Damante, Diego Russo, Stefania Bulotta, Valentina Maggisano

**Affiliations:** 1 Department of Health Sciences, University of Catanzaro ‘Magna Graecia’, 88100 Catanzaro, Italy; francesca.capriglione@hotmail.it (F.C.); maiuolo@unicz.it (J.M.); celano@unicz.it (M.C.); vmaggisano@unicz.it (V.M.); 2 Department of Medicine, University of Udine, 33100 Udine, Italy; giuseppe.damante@uniud.it

**Keywords:** endocrine disruptors, normal thyroid cells, cadmium, quercetin, ROS

## Abstract

Various natural compounds have been successfully tested for preventing or counteracting the toxic effects of exposure to heavy metals. In this study, we analyzed the effects of cadmium chloride (CdCl_2_) on immortalized, non-tumorigenic thyroid cells Nthy-ori-3-1. We investigated the molecular mechanism underlying its toxic action as well as the potential protective effect of quercetin against CdCl_2_-induced damage. CdCl_2_ suppressed cell growth in a dose- and time-dependent manner (IC50 value ~10 μM) associated with a decrease in levels of phospho-ERK. In addition, CdCl_2_ elicited an increase in reactive oxygen species (ROS) production and lipid peroxidation. A significant increase in GRP78, an endoplasmic reticulum (ER) stress-related protein, was also observed. Supplementation of quercetin counteracted the growth-inhibiting action of CdCl_2_ by recovering ERK protein phosphorylation levels, attenuating ROS overproduction, decreasing MDA content and reducing the expression of GRP78 in cells exposed to CdCl_2_. Thus, in addition to revealing the molecular effects involved in cadmium-induced toxicity, the present study demonstrated, for the first time, a protective effect of quercetin against cadmium-induced damages to normal thyroid cells.

## 1. Introduction

Cadmium (Cd) is one of the most toxic environmental pollutants derived from natural sources as well as human activities. Contaminated air, water, food and cigarette smoke are the major sources of human Cd exposure [1]. Because of its extremely long biological half-life and low excretion rate, Cd may accumulate inside the body, causing a broad range of cytotoxic effects, especially to the kidneys, liver and muscles [2,3]. The presence of cysteine-rich proteins containing thiol groups able to bind Cd makes even the thyroid gland a potential site of Cd deposition, and it has been reported that Cd blood concentration correlates positively with its accumulation in thyroid tissue [4,5]. In accordance, in a study conducted by Uetani et al. [6] in people living in a Cd-polluted area of Japan, Cd concentrations in the thyroid gland were three times higher compared to those residing in non-polluted areas [6]. Although Cd thyrotoxicosis has been widely documented, most works have focused on thyroid hormone function, yielding conflicting data [4,5]. Few studies have investigated the action of Cd on human thyroid cells and the underlying molecular and cellular mechanisms involved in thyroid toxicity, and most studies have been performed in cancer cell lines [7,8,9].

Modulation of cellular redox status via indirect increase in reactive oxygen species (ROS) levels (i.e., decreased concentration of intracellular antioxidants and reduced activity of antioxidant enzymes) and mitochondrial damage represents one of the main mechanisms of Cd-induced cytotoxicity [4,10]. Various antioxidant compounds present especially in edible vegetables have been successfully tested for preventing or counteracting the toxic effects of exposure to heavy metals including Cd [4,10,11]. In this context, the flavonoid quercetin is considered a very promising potential therapeutic agent for its strong antioxidant activity exerted by scavenging free radicals and chelating transition metal ions responsible for the generation of ROS [12]. Indeed, various reports described the protective effect of quercetin against Cd-induced cytotoxicity by attenuating lipid peroxidation and increasing intracellular antioxidant action [13,14,15].

In the present study, we analyzed the effects of cadmium chloride (CdCl_2_) on non-tumorigenic human thyroid cells and the molecular mechanism associated with its toxic action. Moreover, we investigated the effects of quercetin against CdCl_2_-induced damage in thyroid cells. Since studies on Cd blood and thyroid concentrations in humans produced complex and often conflicting results [4,5], the experimental design adopted in our study included dose- and time-response experiments in order to define Cd concentration.

## 2. Results

### 2.1. Effects of CdCl_2_ Alone or in Combination with Quercetin on Thyroid Cell Proliferation

**Figure 1:**
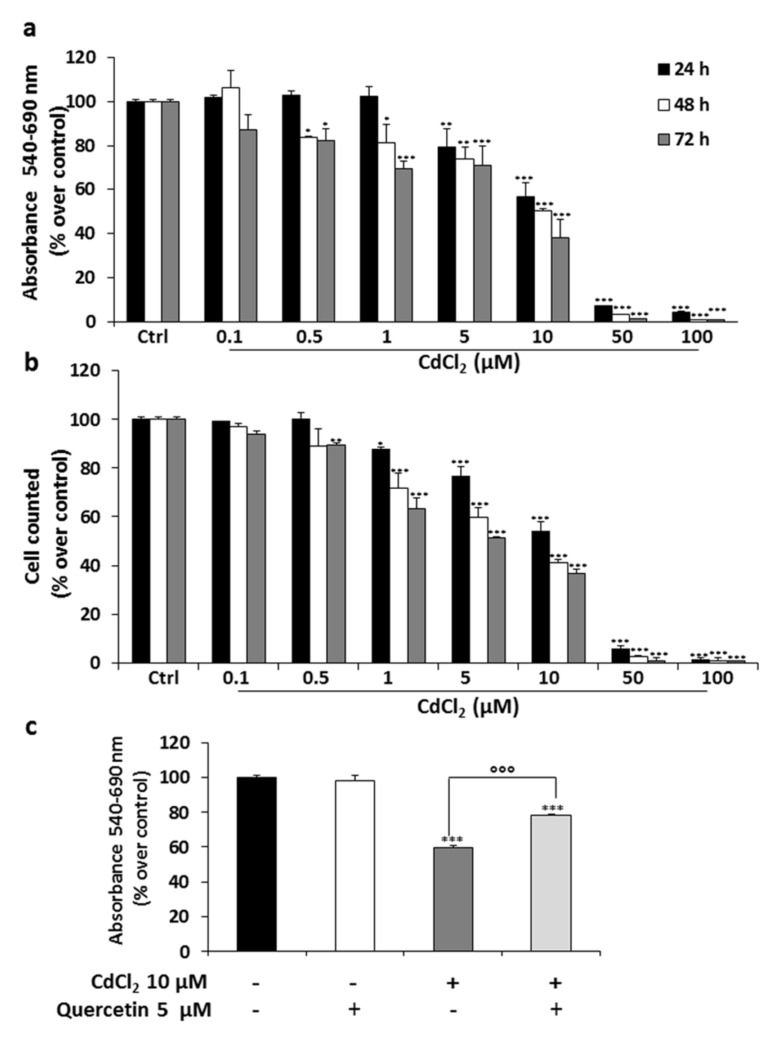
(**a**,**b**) Effects of treatment with CdCl_2_ on thyroid cell viability. Nthy-ori-3-1 cell viability was measured by MTT and cell count assays after 24, 48 and 72 h of treatment with various concentrations of CdCl_2_. (**c**) Effects of quercetin on viability of Nthy-ori-3-1 cells treated with CdCl_2_. Quercetin and CdCl_2_ were administrated alone or in combination, and cell viability was analyzed by MTT assay after 24 h of treatment. Results are expressed as percentage of cell viability reduction vs. untreated cells (Ctrl) (panels **a** and **b**) or vehicle (panel **c**), representing the mean ± SD of three independent experiments. Statistical analysis was performed by using the Tukey–Kramer multiple comparisons test. * *p* < 0.05, ** *p* < 0.01, *** *p* < 0.001 vs. Ctrl, °°° *p* < 0.001 vs. CdCl_2_ 10 μM.

We first evaluated the effect of exposure of Nthy-ori-3-1 thyroid cells to various concentrations of CdCl_2_ (0.1, 0.5, 1, 5, 10, 50 and 100 μM) for 24, 48 and 72 h. By MTT assay, we observed a time- and concentration-dependent decrease in cell proliferation (Figure 1a). After 24 h of treatment, a reduction in cell growth was evident at 10 μM (IC50, *p* < 0.001), and a stronger toxic effect of CdCl_2_ was observed after 72 h of treatment (~70% reduced number over control, *p* < 0.001). The exposure to higher concentrations of CdCl_2_ (50 μM and 100 μM) caused full inhibition of cell viability. Similar results were obtained by cell counting (Figure 1b). Since it has been reported that the flavone quercetin is able to prevent several damages caused by heavy metals, we tried to reverse the cytotoxic effect of CdCl_2_ by using this natural compound, dissolved in DMSO (control) at the concentration of 5 μM. The addition of quercetin in the cells treated with CdCl_2_ 10 μM counteracted the growth-inhibiting action of CdCl_2_ (~20% over CdCl_2_ alone, after 24 h of treatment, *p* < 0.001) (Figure 1c). Similar effects were obtained even after 48 and 72 h of co-administration of both compounds (data not shown).

### 2.2. Modulation of the ERK Pathway in Thyroid Cells by CdCl_2_ and Effect of Quercetin

**Figure 2:**
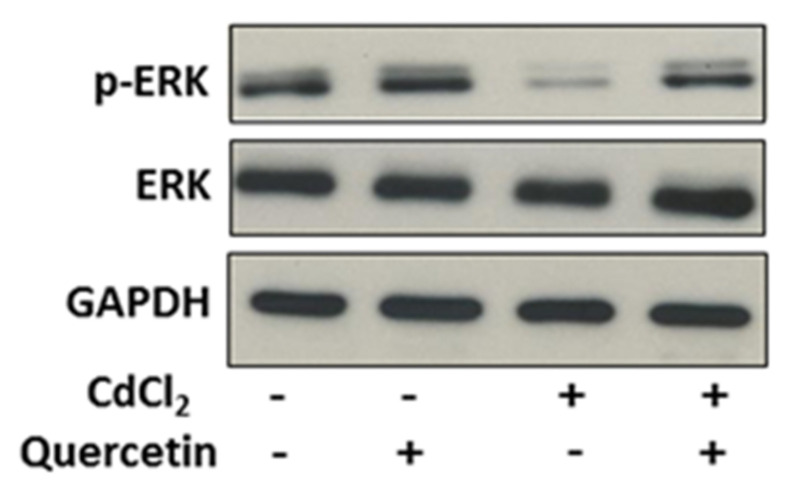
Effects of CdCl_2_ and quercetin on ERK phosphorylation. Nthy-ori-3-1 cells were treated for 3 h with CdCl_2_ 10 μM or/and quercetin 5 μM as described in the Methods. Immunoblot analysis of phosphorylated ERK (p-ERK) and total form of the enzyme (ERK) was performed by Western blotting. GAPDH was used as loading control. An immunoblot representative of three experiments is shown.

To investigate the molecular mechanism involved in the toxic effects of CdCl_2_ in thyroid cells and the interfering action of quercetin, we analyzed the effects of short exposure to these compounds on one of the main signal transduction pathways involved in thyroid cell proliferation. In Nthy-ori-3-1 cells, treatment with CdCl_2_ 10 μM for 3 h resulted in a reduction in ERK phosphorylation without modifying the total levels of this protein (Figure 2). Supplementation of quercetin 5 μM resulted in the recovery of ERK protein phosphorylation levels.

### 2.3. Analysis of the Production of ROS in Thyroid Cells Exposed to CdCl_2_ and Effect of Quercetin

**Figure 3:**
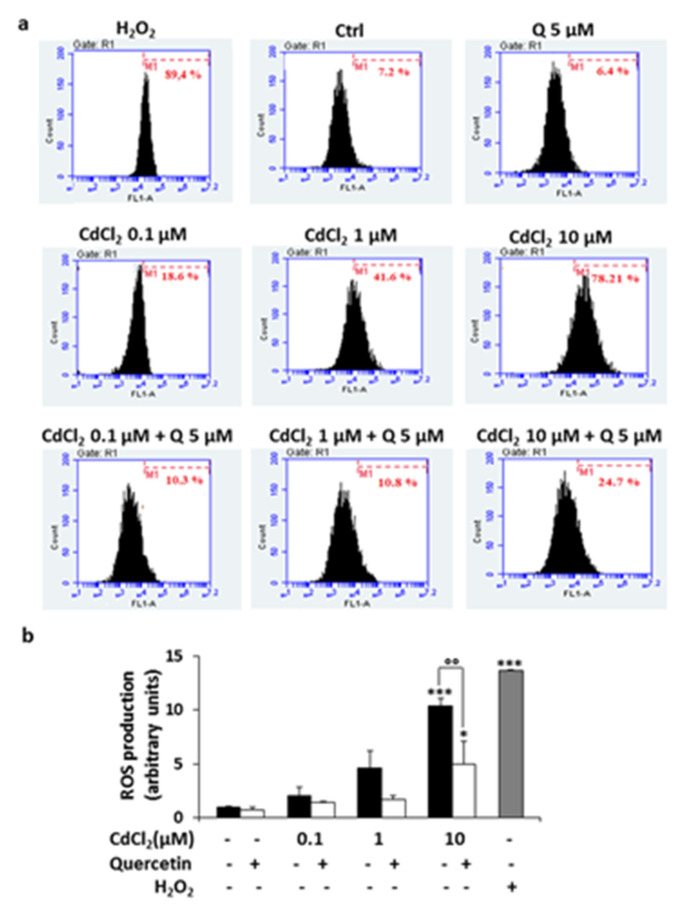
ROS determination in Nthy-ori-3-1 exposed to CdCl_2_ and quercetin. Cells were pretreated for 24 h with or without quercetin (Q) 5 μM and then exposed for 2 h at the indicated concentrations of CdCl_2_; after incubation with H2DCF-DA, the fluorescence of DCF was evaluated by flow cytometry. In panel (**a**), cytofluorimetric plots of a representative experiment of three separate determinations are shown. Panel (**b**) shows the ROS production expressed as fold of increase with respect to untreated cells (Ctrl) considered arbitrarily as 1. H_2_O_2_ (150 μM) was used as the internal positive control. The results represent the mean ± SD of three independent experiments. Statistical analysis was performed using the Tukey–Kramer multiple comparisons test. * *p* < 0.05, *** *p* < 0.01 vs. Ctrl; °° *p* < 0.01 vs. CdCl_2_ 10 μM.

Many reports indicate that the production of ROS is enhanced by exposure to various heavy metals including Cd. Thus, we examined by flow cytometry the effect of CdCl_2_ on ROS production in Nthy-ori-3-1 cells. After 2 h of exposure (Figure 3), the resulting ROS levels had enhanced approximately 10-fold in cells treated with CdCl_2_ 10 μM compared to untreated cells (*p* < 0.001 vs. control). This effect was attenuated when the cultures were pre-treated for 24 h with quercetin 5 μM, as revealed by a leftward shift of fluorescence (*p* < 0.01 vs. CdCl_2_ 10 μM) (Figure 3a,b).

### 2.4. Effect of CdCl_2_ on Lipid Peroxidation and Endoplasmic Reticulum (ER) Stress

**Figure 4:**
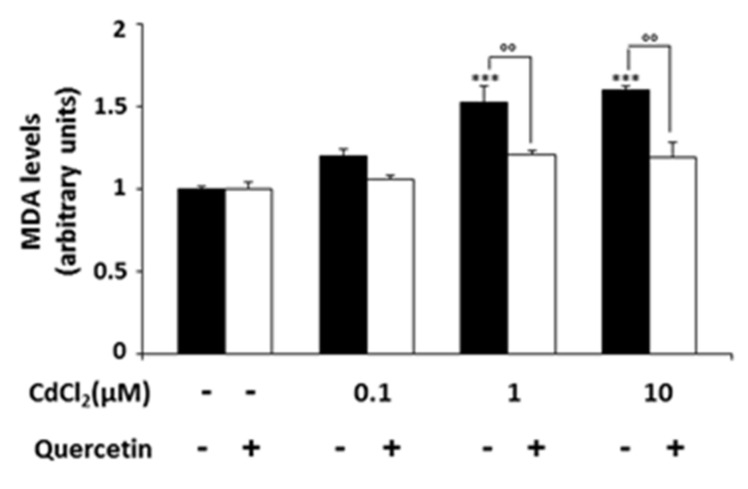
Lipid peroxidation in Nthy-ori-3-1 exposed to CdCl_2_ and quercetin 5 μM. Lipid peroxidation was evaluated by MDA assay (see Materials and Methods) after incubation of cells with quercetin and CdCl_2_ alone or in combination for 24 h. MDA levels were expressed as fold of increase with respect to untreated cells considered arbitrarily as 1. The results are means ± SD of two independent experiments. Statistical analysis was performed using the Tukey–Kramer multiple comparisons test. *** *p* < 0.001 vs. Ctrl; °° *p* < 0.01 vs. CdCl_2_ 1 μM and 10 μM.

Malonildialdehyde (MDA) is a biomarker of oxidative damage, and its levels are considered as a lipid peroxidation index. Thus, to investigate the effect of CdCl_2_ on lipid peroxidation, we measured MDA levels in cells treated for 24 h with CdCl_2_ 0.1, 1 or 10 μM alone or in combination with quercetin 5 μM. As shown in Figure 4, MDA production was increased significantly after incubation with CdCl_2_ 1 and 10 μM compared with untreated cells (*p* < 0.001). This effect was significantly attenuated when the cultures were supplemented with quercetin (*p* < 0.01 vs. CdCl_2_ 1 and 10 μM).

**Figure 5:**
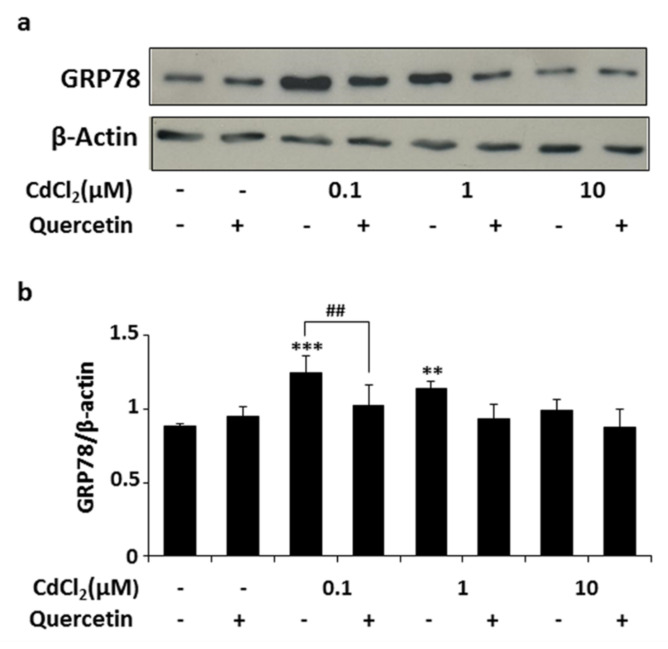
(**a**) Effects of quercetin on ER stress marker GRP78. Western blot analysis was performed on Nthyori-3-1 cells exposed to CdCl_2_ and quercetin (5 μM) alone or in combination for 2 h, as described in the Methods. β-Actin was used as loading control. An immunoblot representative of three experiments is shown. (**b**) Densitometric analysis of GRP78 normalized to β-Actin. Values are expressed as means ± SD from three experiments. Statistical analysis was performed using the Tukey–Kramer multiple comparisons test. ** *p* < 0.01, *** *p* < 0.001 vs. control, ## *p* < 0.01 vs. CdCl_2_ 0.1 μM.

Finally, we evaluated the expression of GRP78, an ER stress-related protein [16], by Western blotting analysis (Figure 5). A significant increase in GRP78 protein expression was detected in Nthy-ori-3-1 cells exposed to 0.1 or 1 μM of CdCl_2_ for 2 h compared with untreated cells. Again, this action was reversed by pretreatment with quercetin 5 μM for 24 h (Figure 5). No differences were observed in cells exposed to Cd 10 μM, probably because at this concentration Cd exerts its cytotoxic effect and ER fails to overturn the stressful conditions [17].

## 3. Discussion

Cd has been identified as an endocrine-disrupting chemical for the thyroid gland [18]. Indeed, it has been reported that Cd exposure determined imbalanced plasma of T3, T4 and TSH levels in in vivo experimental models and in humans, although these data are often contradictory depending on the experimental models and the used dosage [5]. Moreover, other signs markers of Cd thyrotoxicity are histological changes detected in the gland appearing with desquamation of follicular cells and an increase in fibrous tissue and monocyte infiltration in the stroma of the thyroid [4]. Several mechanisms have been proposed to explain Cd cytotoxicity [3], but they have never been evaluated directly in non-tumor thyroid cells. In the present study we used Nthy-ori-3-1 cells, widely used as preclinical model of non-tumorigenic human thyrocytes, to analyze the molecular alterations caused by CdCl_2_ involved in its toxic activity, focusing our attention on the alterations of cellular redox status regulation. Our findings demonstrate that CdCl_2_ suppresses cell growth in a dose- and time-dependent manner and is associated with a decrease in the levels of phospho-ERK, suggesting the involvement of the MAPK-mediated pathway, a well-established transduction pathway of regulation of thyrocyte proliferation [19], and indicated as a target of Cd toxic action even in other cell types [20,21]. In addition, in our experimental model, CdCl_2_ elicited an increase in ROS levels, lipid peroxidation and ER stress, suggesting that the toxic effect of Cd in thyroid cells may be determined by the production of free radicals which, by stimulating lipid peroxidation in biomembranes, lead to cell damage, as also documented by the up-regulation of GRP78, a regulator of ER stress.

More interestingly, our findings demonstrate, for the first time, that the toxic action of Cd on thyroid cells may be counteracted by the addition of quercetin, a bioflavonoid widely distributed in vegetables and fruits known to have an extended spectrum of beneficial effects on human health [22]. There are many reports that have described the ability of this compound to mitigate the oxidative stress effects of various toxic compounds through acting mainly as a ROS scavenger [12,13,14,23,24]. Other experimental evidence has suggested that the cellular effects of polyphenols could be mediated by their interactions with specific proteins of intracellular signaling cascades, including the MAPK pathways [25]. Therefore, it was not surprising to detect a protective effect of quercetin against Cd-induced oxidative damage in thyroid cells. Indeed, we found that supplementation of quercetin counteracted the growth-inhibiting action of Cd, recovering ERK protein phosphorylation levels and attenuating ROS overproduction. The protection against oxidative stress damage was also confirmed by the decrease in lipid peroxidation observed in the cells exposed to Cd, as already shown in other cell models [13,22], as well as by the reduction in the expression of the ER resident chaperone GRP78, involved in the ER stress response determined by ROS generation [16,20,26,27].

Recently, while our study was in progress, Yang et al. [28] described a protective action against Cd thyrotoxicity exerted by fucoxanthin, a powerful antioxidant nutraceutical, confirming the validity of using natural antioxidants as a promising approach to fight against this heavy metal-induced toxicity. Further studies, including the investigation of chronic exposure of thyroid cells to mixtures of pollutants, will allow us to better characterize the effects of the protective capacity of nutraceuticals against this endocrine disruptor.

## 4. Materials and Methods

### 4.1. Chemicals

CdCl_2_, dimethyl sulfoxide (DMSO), 2′,7′-dichlorodihydrofluorescein diacetate (H2DCF-DA), glacial acetic acid, 3-(4,5-dimethylthiazol-2-yl)-2,5-diphenyltetrazolium bromide (MTT) and quercetin were purchased from Merck Serono (Rome, Italy). RPMI 1640 and phenol red-free RPMI were purchased from Thermo Fisher Scientific Inc. (Waltham, MA, USA). Antibodies anti-ERK and anti-phospho-ERK were obtained from Santa Cruz Biotechnology (DBA, Segrate, Milan, Italy); anti-β-actin and anti-GAPDH were obtained from Merck Serono (Rome, Italy), and anti-GRP78 was purchased from BD (Milan, Italy).

### 4.2. Cell Culture and Cell Proliferation Assays

Nthy-ori-3-1 thyroid cells, purchased from the European Collection of Authenticated Cell Culture (ECACC, Public Health England, Porton Down, Salisbury, SP4 0JG, UK), are human non-tumorigenic thyroid cells that express the thyroglobulin gene, but not thyroperoxidase, and express only very low levels of both the TSH receptor and NIS gene [29]. The cells were cultured in RPMI 1640 and maintained at 37 °C in a humidified 5% CO_2_ [29]. In the experiment, cells were seeded in 12- or 96-well plates at a density of 50 × 10^3^ and 6 × 10^3^, respectively. After 24 h the growth medium was replaced by fresh normal medium supplemented with various doses of CdCl_2_ ranging from 0.1 to 100 μM, quercetin 5 μM, or its vehicle (DMSO) alone at a non-toxic concentration (below 10% *v*/*v*) [30]. After 24, 48 and 72 h in CdCl_2_, quercetin alone or in combination with CdCl_2_ 10 μM, cell proliferation was assessed by cell count or MTT assay [31,32]. For cell counting, after treatment with CdCl_2_, viable cells were counted in chamber slides (Countess, automated cell counter, Thermo Fisher Scientific) after Trypan blue staining. For the MTT assay, a microplate spectrophotometer (xMark, Biorad, Milan, Italy) at a wavelength of 540 nm and a reference wavelength of 690 nm was used to quantify the solubilized product. Results are expressed as percentages over untreated cells or DMSO (control), considered equal to 100 for each time (24, 48 and 72 h).

### 4.3. Western Blot Analysis

Twenty micrograms of total proteins, extracted as previously described [31], was run on a 9 or 12% SDS PAGE gel, transferred to PVDF membranes (VWR, Milan, Italy), blocked with TTBS/milk (TBS, 1% Tween 20 and 5% non-fat dry milk) and incubated overnight with anti-ERK antibody diluted 1:100 or anti-phospho-ERK antibody diluted 1:500, anti-GRP78 antibody diluted 1:2000, and anti-β-actin or anti-GAPDH antibody diluted 1:5000. The membranes were incubated with horseradish peroxidase-conjugated antibody (Transduction Laboratories, Lexington, KY, USA) in TTBS/milk. The Western blot detection system ECL Plus (Perkin Elmer, Monza, Italy) was used to visualize the proteins.

### 4.4. ROS Assay

ROS were measured as described by Lepore et al. [33]. Nthy-ori-3-1 cells were pre-treated or not for 24 h with quercetin 5 μM and then exposed for 2 h to CdCl_2_ 0.1, 1 and 10 μM. Subsequently, the cells were washed with PBS, trypsinized, resuspended in phenol red-free RPMI medium containing a fluorescent probe H2DCF-DA (25 μM) and cultured for 30 min at 37 °C. ROS production was evaluated by flow cytometric analysis using the flow cytometer Accuri C6 (Becton Dickinson, San Jose, CA, USA). Hydrogen peroxide (H_2_O_2_) 150 μM was used as an internal positive control.

### 4.5. MDA Assay

Lipid peroxidation was evaluated by measuring the reaction products of MDA with thiobarbituric acid (TBA), which form a colorimetric product proportional to the MDA present. The cells were placed in 10 cm diameter cell culture Petri dishes and, the following day, were incubated for 24 h with medium containing CdCl_2_ 0,1, 1 and 10 μM alone or in combination with quercetin 5 μM. At the end of the treatment, cells were scraped, and cell suspension was subjected to a freezing/thawing cycle. Subsequently, 36 mM TBA dissolved in glacial acetic acid was added and the mixture heated for 60 min to 100 °C. The reaction was interrupted by placing the vials on ice for 10 min, and a spectrophotometric reading was carried out at 532 nm.

### 4.6. Statistical Analysis

The results are expressed as means ± standard deviation (SD). Statistical analysis was conducted using GraphPad Prism version 5.0 statistical software (GraphPad Software Inc., San Diego, CA, USA). Comparisons among multiple groups were performed by one-way ANOVA followed by the Tukey–Kramer test.

## 5. Conclusions

In summary, in this work we demonstrate that CdCl_2_ suppresses thyroid cell viability in vitro by inhibiting the ERK1/2 pathway, inducing ROS generation and lipid peroxidation and determining ER stress. We also found that addition of quercetin was able to counteract Cd-toxic effects, encouraging the use of this nutraceutical for blocking the action of this disruptor.

## Data Availability

Not applicable.
